# Detection of drugs of abuse: evolving analytical strategies and emerging challenges

**DOI:** 10.3389/fchem.2026.1739599

**Published:** 2026-08-03

**Authors:** Nagwa Eliw, Manal A. Alhefeiti, Ahmed A. Shamroukh, Iltaf Shah

**Affiliations:** 1 Department of Chemistry, College of Science, UAE University, Al Ain, United Arab Emirates; 2 Forensic Science Laboratory, Ministry of Justice, Al Ain, United Arab Emirates; 3 Department of Chemistry, Faculty of Science, Qena University, Qena, Egypt

**Keywords:** analytical techniques, drugs of abuse, forensic toxicology, mass spectrometry, new psychoactive substances (NPS), portable sensors

## Abstract

The detection of drugs of abuse in biological and non-biological matrices remains a cornerstone of forensic, clinical, and public health investigations. Traditional matrices such as urine and blood provide short-term evidence of drug intake, while alternative specimens such as hair offer extended detection windows, and oral fluid provides a reliable indicator of recent drug intake comparable to blood. This review summarizes current knowledge on specimen selection, factors influencing drug absorption, distribution, and elimination, and highlights analytical strategies ranging from chromatographic and mass spectrometric methods to emerging electrochemical, optical, and biosensor platforms. Special emphasis is placed on the challenges posed by new psychoactive substances (NPS) such as synthetic cannabinoids (SCs), cathinones, hallucinogens, and amphetamine type stimulants, which continuously evolve to evade regulation and complicate detection. Advances in sample preparation, miniaturization, and portable analytical devices are critically evaluated in terms of sensitivity, specificity, and applicability to forensic and clinical settings. A central theme of this review is the two-tiered analytical framework that integrates rapid field screening technologies with highly selective chromatographic–mass spectrometric confirmation, providing a practical and scalable approach for addressing both conventional drugs of abuse and emerging NPS challenges.

## 1 Introduction

The use and abuse of psychoactive substances constitute a persistent and evolving global challenge, with documented historical use spanning millennia. While certain drugs offer legitimate therapeutic benefits, their non-medical misuse presents profound public health, socioeconomic, and clinical dilemmas (Mali et al., 2011). Drug addiction is a chronic, relapsing condition that can lead to severe physiological consequences, including neurotoxicity, cardiovascular complications, immune system impairment, and mortality. The United Nations Office on Drugs and Crime (UNODC) 2023 World Drug Report underscores the scale of this issue, indicating that over 296 million people used drugs globally in 2021, a 23% increase over the previous decade (Scanferla et al., 2022).

The accurate detection and identification of drugs of abuse are fundamental pillars supporting forensic toxicology, clinical diagnostics, occupational health screening, and criminal justice proceedings. The landscape of substance abuse is dominated by several classes of drugs, most notably opioids, cocaine, cannabis, amphetamine-type stimulants (ATS), and a rapidly expanding cohort of NPS. These NPS, which include SCs, cathinones, and hallucinogenic analogs, are deliberately designed to mimic the effects of controlled drugs while evading legal restrictions and standard detection methodologies, thereby complicating analytical efforts (Zhang et al., 2025).

The choice of biological matrix is a critical factor in toxicological analysis, directly influencing the detection window and interpretive value of results. Traditional matrices such as blood and urine provide short-term evidence of drug intake, with blood concentrations often correlating with toxicological and pharmacological effect and urine offering ease of collection. In contrast, alternative specimens like hair, OF, and sweat have gained prominence for their extended detection windows (weeks to months in the case of hair), resistance to adulteration, and non-invasive collection protocols (AGJTdm, 2004; KINTZ PJFsi, 2008; Samyn and CJIjolm, 2000). However, each matrix presents unique challenges concerning drug incorporation mechanisms, stability, and the interpretation of quantitative results. A comparison of various common biological matrices for drug detection is shown in Table 1.

**TABLE 1:** Comparison of common biological matrices for drug detection.

Matrix	Detection window	Key advantages	Key disadvantages	Primary analytical methods
Blood (Plasma/Serum)	Hours–1–2 days	Correlates with pharmacological effects; well-established interpretation	Invasive; short detection window; requires skilled personnel	LC–MS/MS, GC–MS, Immunoassay
Urine	1–3 days (longer for chronic use)	Non-invasive; high drug/metabolite concentration; widely used	Easily adulterated; poor correlation with impairment	Immunoassay, LC–MS/MS, GC–MS
OF	24–48 h	Non-invasive; reflects recent use; difficult to adulterate	Low volume; variability; contamination risk	LC–MS/MS, Immunoassay, Biosensors
Hair	Weeks–months	Long detection window; resistant to adulteration; exposure history	Cannot detect recent use; contamination risk; complex prep	LC–MS/MS, GC–MS
Sweat	Hours–weeks	Non-invasive; continuous monitoring possible	Variable secretion; contamination risk; limited protocols	LC–MS/MS, Immunoassay

In response to these analytical challenges, a diverse arsenal of techniques has been developed. Immunoassays provide rapid screening but are often plagued by cross-reactivity and provide only qualitative results, necessitating confirmatory analysis. Chromatographic techniques coupled with mass spectrometry, such as liquid chromatography-tandem mass spectrometry (LC-MS/MS) and gas chromatography-mass spectrometry (GC-MS), represent the gold standard for sensitive, specific, and multi-analyte quantification (O'Halloran, 1993). Concurrently, the field is witnessing significant innovation in the development of portable, rapid detection platforms. Emerging electrochemical, optical, and biosensor technologies promise on-site analysis capabilities, though they often face hurdles in sensitivity, validation, and matrix interference compared to established laboratory methods.

Therefore, this comprehensive review seeks to critically synthesize and evaluate the current state of analytical strategies for the detection of drugs of abuse. It specifically aims to:

1. Provide a systematic overview of the most prevalent and challenging drugs and drug classes, with a dedicated focus on LSD, synthetic cathinones, SCs, psilocybin, MDMA, and methamphetamine (MA).
2. Critically compare and contrast traditional laboratory based chromatographic and spectrometric methods with emerging portable sensor-based platforms, delineating their respective advantages, limitations, and specific applications in forensic and clinical settings.
3. Identify persistent analytical challenges such as the continuous emergence of NPS, matrix effects, and the need for green chemistry principles and discuss future directions for the field, including the integration of advanced materials, multiplexed systems, and point-of-care technologies.

By addressing these objectives, this review aims to provide a valuable resource for forensic scientists, clinical toxicologists, and researchers, facilitating informed decisions on method selection and highlighting the trajectory of innovation in drug detection.

The detailed systematic literature search strategy, inclusion/exclusion criteria, and quality assessment framework are provided in the Supplementary Material (ESI).

## 2 Overview of selected popular drugs of abuse

### 2.1 LSD (lysergic acid diethylamide)

**FIGURE 1:**
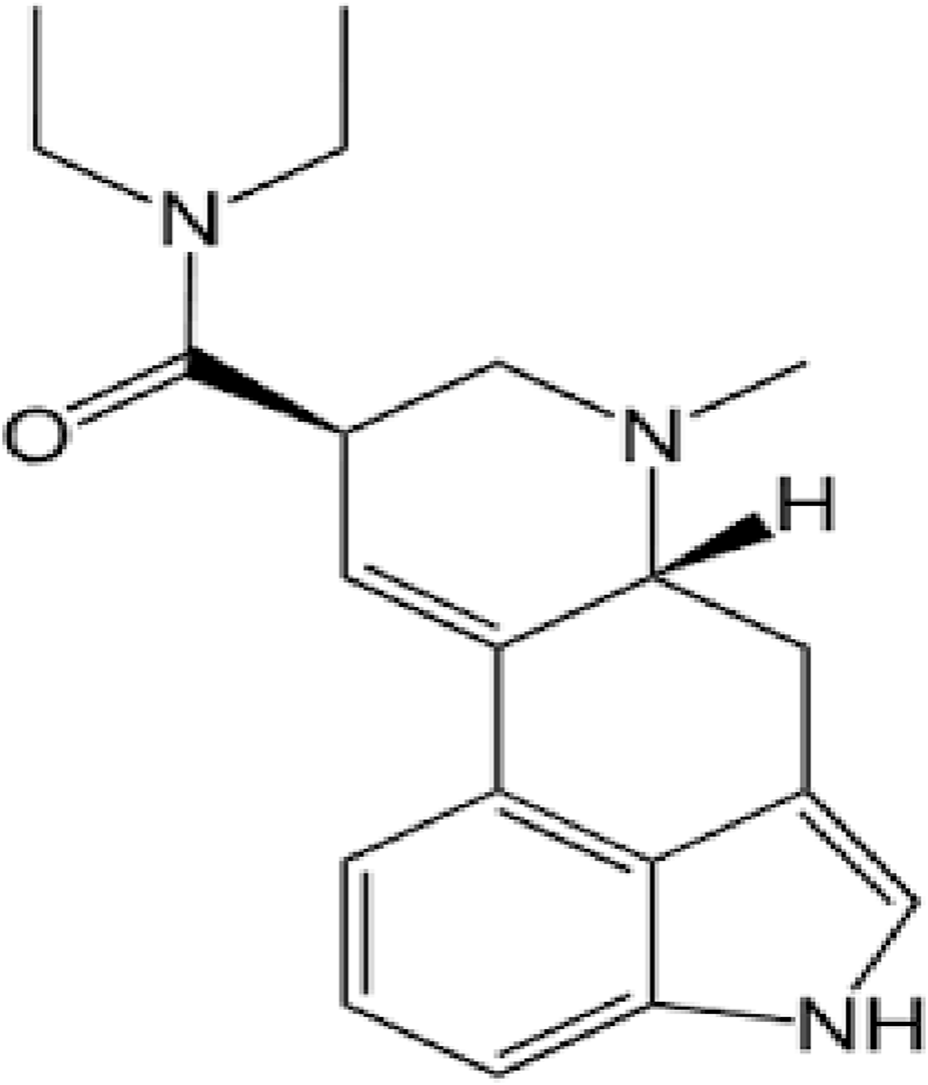
Lysergic acid diethylamide.

Seizures of both traditional and new psychoactive drugs, such as LSD, have grown worldwide in recent years. Claviceps purpurea produces LSD, a highly strong hallucinogenic chemical and semi-synthetic derivative of lysergic acid (Figure 1). Its forensic importance has resurfaced as the usage of LSD has increased and structurally comparable lysergamide analogs have developed in the NPS market (Brandt et al., 2016; Libanio Osorio Marta RFJDmr, 2019). LSD causes powerful hallucinogenic effects principally through its interaction with serotonin (5-HT) receptors. It is typically taken orally; however additional routes of exposure have been described. Despite its low incidence in comparison to other illegal chemicals, LSD is nonetheless toxicologically relevant due to its intense psychedelic effects and occasional link with severe negative consequences (Fysh et al., 1985; Klock et al., 1975; O'Halloran, 1993). LSD is most commonly administered via blotter paper, where the drug is deposited onto absorbent material and placed on or under the tongue. In this form, sublingual absorption represents a major route of administration, allowing rapid uptake through the oral mucosa and bypassing, to some extent, first-pass metabolism. In addition to sublingual use, LSD may also be swallowed, leading to gastrointestinal absorption; however, this route may result in slightly delayed onset of effects. Less commonly, LSD can be encountered in liquid form or gelatin preparations.

LSD poses significant analytical issues due to its extremely low concentrations in biological matrices (often in the sub-ng/mL range), fast metabolism, and structural similarities to developing analogs. Furthermore, it is susceptible to deterioration under light and temperature conditions, complicating detection and quantification. These properties need the employment of extremely sensitive and selective analytical methods, notably LC-MS/MS and high-resolution mass spectrometry, for accurate identification in forensic and clinical toxicology. The analytical detection of LSD is challenging because of its ultra-trace concentrations in biological matrices, rapid metabolism, chemical instability, and structural similarity to emerging lysergamide analogs. These characteristics necessitate highly sensitive and selective analytical platforms, particularly LC–MS/MS and LC–HRMS, combined with optimized sample preparation strategies.

### 2.2 Synthetic cathinones

**FIGURE 2:**
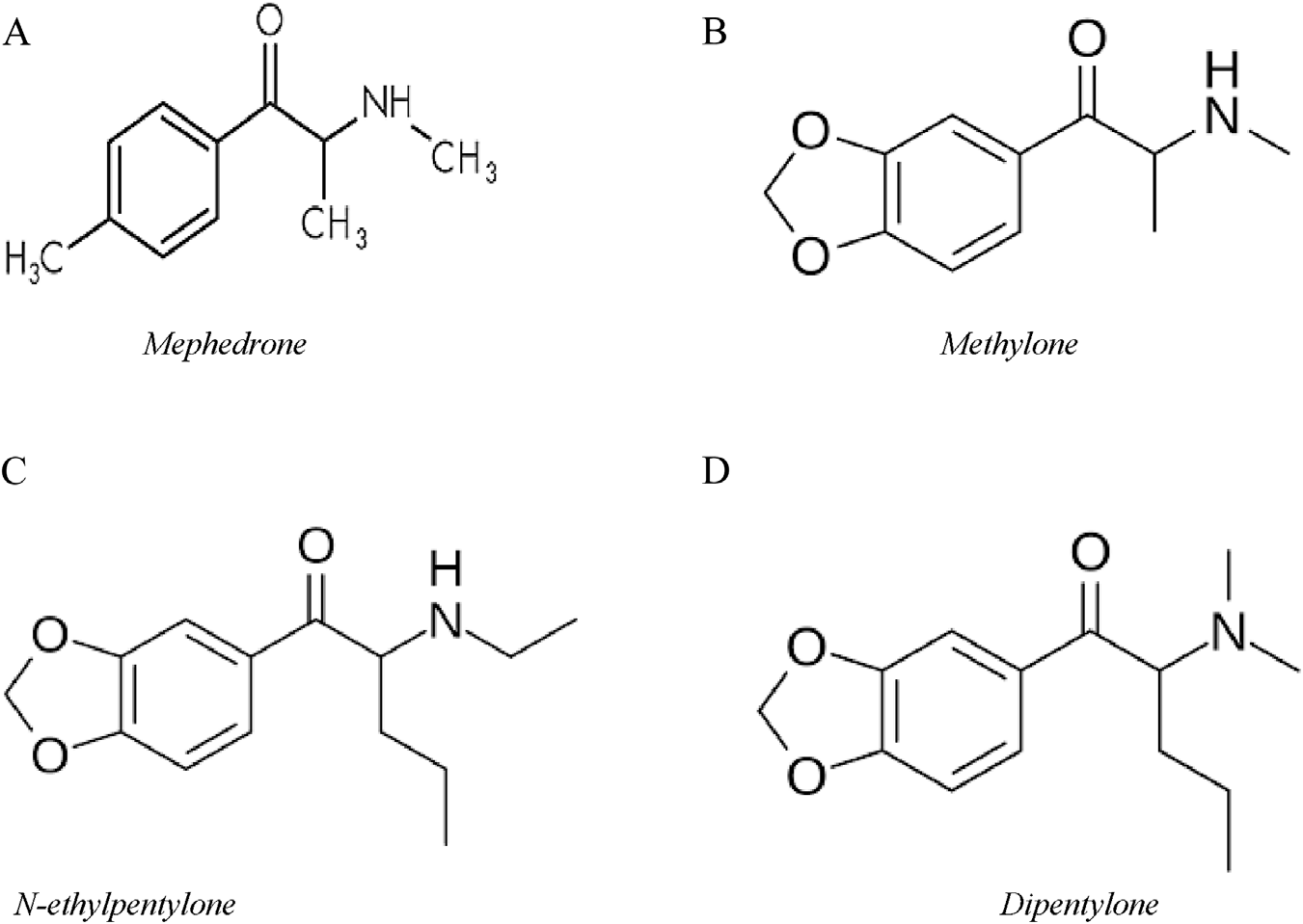
Representative chemical structures of synthetic cathinones **(A)** Mephedrone (4-methylmethcathinone) **(B)** Methylone **(C)** N-ethylpentylone, and **(D)** Dipentylone. These compounds illustrate structural variations in synthetic cathinones, including differences in aromatic ring substitution, alkyl side chains, and amine functionalization, which influence their physicochemical properties, pharmacological activity, and analytical detectability.

Synthetic cathinones, a subset of NPS, are structurally developed from cathinone, the β-keto analogue of amphetamine (Oliver et al., 2019). These molecules are intended to replicate the stimulant effects of traditional drugs like cocaine, amphetamines, and MDMA while avoiding legal oversight through ongoing structural alteration (Prosser and Nelson, 2012; Zawilska and JJFsi, 2013). Common examples include mephedrone (4-methylmethcathinone), methylone, N-ethylpentylone, and dipentylone (Figure 2), which differ in alkyl chain replacements, aromatic ring functionalization, and amine modifications (Zawilska and JJFsi, 2013; Zuang et al., 2021). These structural changes have a substantial impact on their physicochemical characteristics, such as polarity, lipophilicity, and metabolic stability, which directly affect analytical detection and identification.

Toxicologically, synthetic cathinones have been linked to significant side effects such as sympathomimetic toxicity, hyperthermia, agitation, psychosis, and cardiovascular problems. Their structural resemblance to amphetamine-type stimulants challenges both toxicological interpretation and analytical differentiation in screening tests (PJGPTVS, 1984).

Synthetic cathinones provide significant analytical problems due to their structural variety, quick production of novel analogs, and constantly changing chemical profiles (Di Trana et al., 2022; Kar et al., 2015; Prosser and Nelson, 2012; Zawilska and JJFsi, 2013; Zuang et al., 2021). While early compounds like mephedrone and methylone are fairly well understood, subsequent derivatives like N-ethylpentylone and dipentylone sometimes need high-resolution analytical methods for accurate identification (LC-MS/MS) is still the preferred approach for focused quantification, whereas high-resolution mass spectrometry (LC-HRMS) is required for untargeted screening and retrospective identification of new chemicals (Fan et al., 2020; Pascual-Caro et al., 2020). The ongoing development of synthetic cathinones emphasizes the importance of adaptive analytical procedures, often updated spectrum libraries, and comprehensive detection techniques capable of recognizing both recognized and new compounds across different matrices. Synthetic cathinones present considerable analytical challenges owing to their structural diversity, rapid emergence of new analogs, varying polarity, and extensive metabolism.

### 2.3 Synthetic cannabinoids (spice)

**FIGURE 3:**
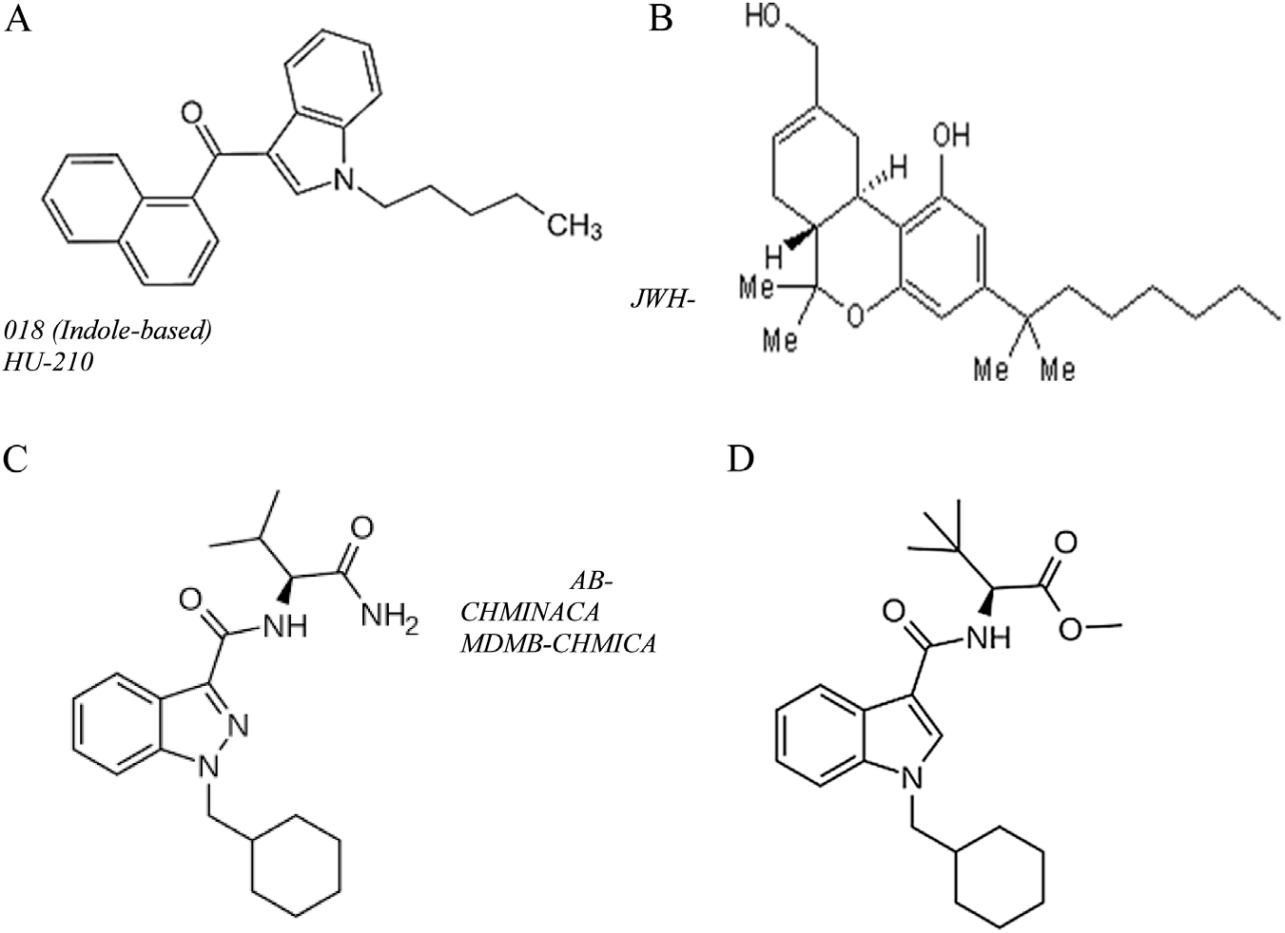
Representative chemical structures of SCs from different generations **(A)** JWH-018 (first-generation, indole-based) **(B)** HU-210 (second-generation, fluorinated analog) **(C)** AB-CHMINACA (third-generation, indazole-based), and **(D)** MDMB-CHMICA (newer generation, ester-containing compound). These examples illustrate the structural evolution of SCs, including variations in core scaffolds, functional groups, and side chains, which contribute to differences in potency, metabolism, and analytical detectability.

SCs are gaining popularity and overtaking traditional cannabis, which is the most often used illicit substance globally. SCs are a highly heterogeneous group of synthetic substances designed to activate the same cannabinoid receptors (CB1 and CB2) as Δ^9^-tetrahydrocannabinol (THC), the active molecule in cannabis, they are marketed as Spice or K2 (Debruyne, 2015). SCs have a wide range of structures because they include many different chemical classes, such as aminoalkylindoles (like the JWH series), classical cannabinoids (like HU-210) (Figure 3), non-classical cyclohexylphenols, quinolinyl esters, and hybrid compounds (Breivogel et al., 2020; Castaneto M. S. et al., 2014). The majority of SCs function as full agonists with binding affinities several times higher than Δ^9^-THC, which is a partial agonist at CB1/CB2 receptors, producing noticeably stronger effects (Tai et al., 2016). SCs may be 30–100 times more potent than THC, according to *in vivo* and clinical data, and even at low dosages, they can cause serious toxic and psychiatric side effects (Alipour et al., 2019; Breivogel et al., 2020; Castaneto M. S. et al., 2014; de Oliveira et al., 2023). Clinically, SC use has been linked to a wide range of acute adverse events that are uncommon or nonexistent in natural cannabis intoxication, including tachycardia, hypertension, chest pain, nausea, seizures, acute kidney injury, psychosis, and muscle breakdown (rhabdomyolysis) (Prete et al., 2025). SCs are often compared to marijuana’s ‘high’, although their toxicity differs significantly. Compared to plant-based marijuana, synthetic cannabis is typically more harmful. Marijuana has modest and short-term negative effects, whereas SCs have been linked to serious repercussions such as hallucinations, psychosis, hypertension, seizures, and panic attacks. Synthetic marijuana has unexpected effects and can have serious and catastrophic consequences for patients (Alves et al., 2020). In the early 2000s, substances (e.g., JWH-018) developed for research became popular in smoking mixes, especially in countries where recreational cannabis use was prohibited or users wanted to avoid detection through standard drug testing. Several SCs were developed in secret labs and offered online as legal alternatives to cannabis (or ‘legal highs’). Known as ‘K2′ in North America, ‘Spice’ in Europe, ‘Youcatan’, ‘Chill’, or ‘Black Mamba’, these mixtures have been widely advertised as smokable herbal combinations, and are allegedly safe for consumption (Spaderna et al., 2013). SCs can cause unconsciousness, psychosis, agitation, and in some cases, death. Their high lipophilicity, extensive metabolism, and structural diversity complicate extraction, identification, and toxicological interpretation, necessitating advanced analytical approaches discussed in Section 3.

### 2.4 Psilocybin (mushrooms)

**FIGURE 4:**
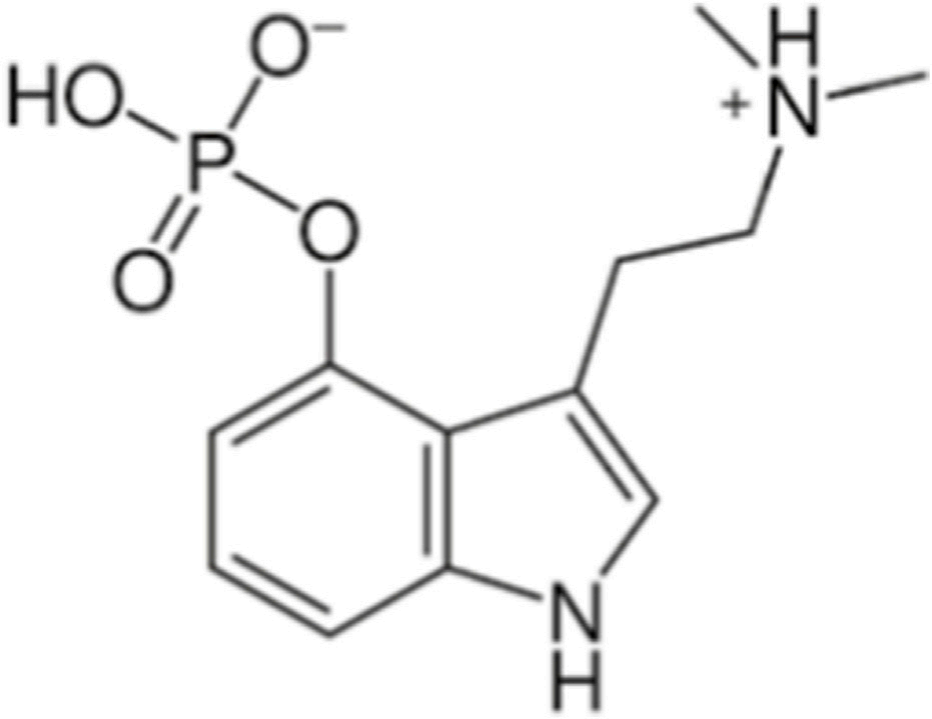
Psilocybin.

A significant number of drugs currently subject to abuse originate from natural sources. Psychedelic mushrooms, often referred to as ““magic mushrooms”“, are known for their hallucinogenic properties, which can profoundly alter an individual’s perception of reality (Goff et al., 2024). Several fungal genera such as Psilocybe, Conocybe, Pluteus, and Panaeolusare known to contain the tryptamine compounds psilocybin and psilocin (Solano et al., 2019; Strauss et al., 2022). These psychoactive substances act primarily on serotonin (5-HT) receptors, leading to a range of sensory and cognitive disturbances, including visual, auditory, and psychological hallucinations (Abbas et al., 2021). Magic mushrooms were used by ancient civilizations for medicine or religious purposes. However, the recreational use of hallucinogenic mushrooms is a relatively new phenomenon. Data show that such misuse has been prevalent since the 1960s (Esteves et al., 2022). In 1971, psilocybin and psilocin were included to the United Nations Convention’s Schedule I of Controlled Substances under the Psychotropic Substances section 35. Magic mushrooms are still used recreationally today, but their prevalence has fluctuated over time. People frequently jumped out of windows under the mushroom cloud. Addiction to mushrooms causes catastrophic brain cell damage. Fly agaric mushrooms are used in Ukraine for treatment, relaxation, and hallucinogenic effects, according to a ““trend”“. Poisonous mushrooms are sometimes used by youngsters and young people for arguing or creating TikTok videos (Shapovalova et al., 2023). In recent years, usage of hallucinogenic mushrooms has been recorded in many nations. Abusers typically consume dried hallucinogenic mushrooms or powdered mushrooms in capsule form. Long-term use of hallucinogenic mushrooms can lead to negative mental health effects and addiction, making it difficult to extricate (Saito et al., 2004). The analytical detection of psilocybin and psilocin is particularly affected by their chemical instability and matrix-dependent behavior. Psilocybin chemical structure is shown in Figure 4.

### 2.5 MDMA

**FIGURE 5:**
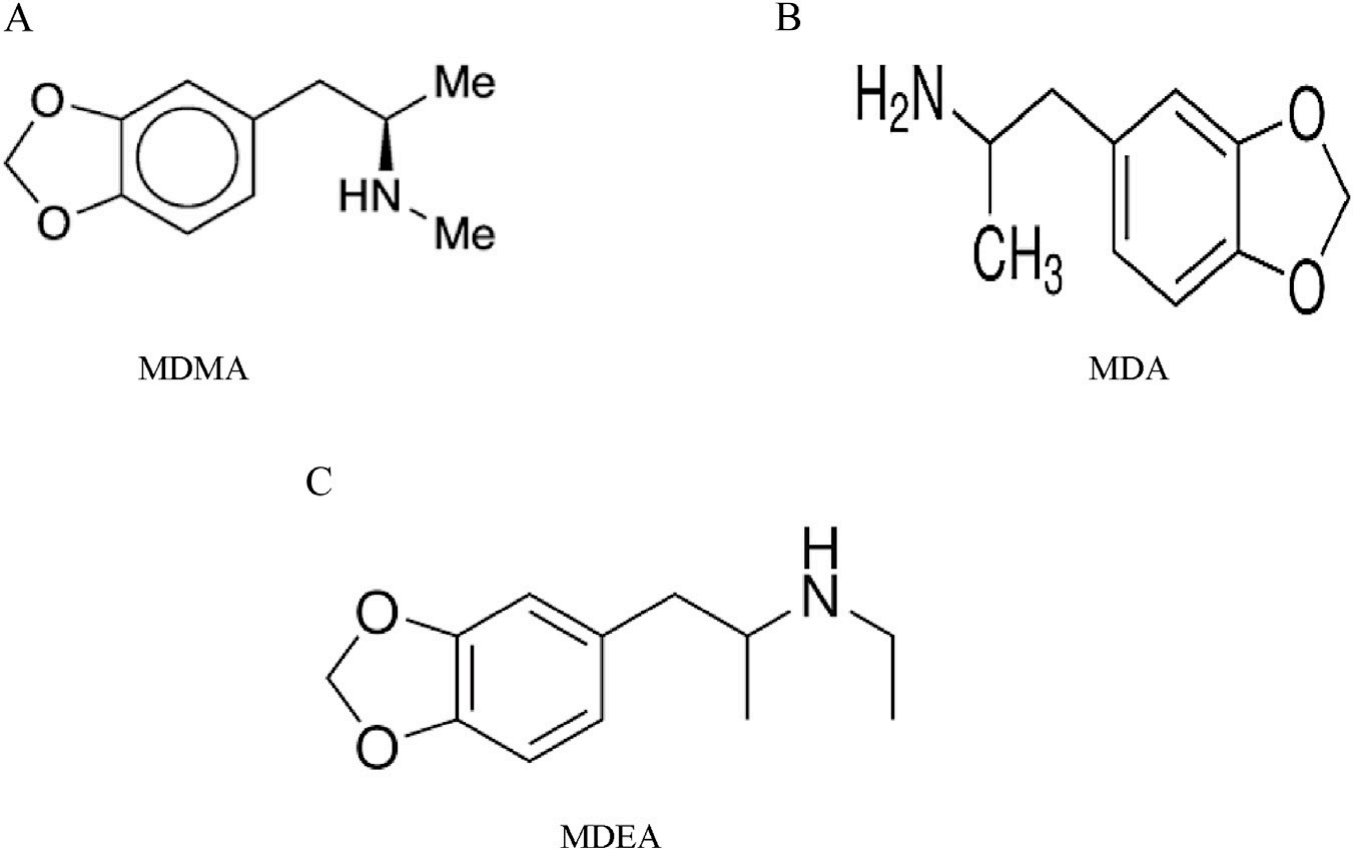
Chemical structures of MDMA and its analogues **(A)** MDMA (3,4-methylenedioxymethamphetamine) **(B)** MDA (3,4-methylenedioxyamphetamine), and **(C)** MDEA (3,4-methylenedioxyethylamphetamine). These compounds share a common methylenedioxy-substituted aromatic ring but differ in their side-chain substitutions, which influence their pharmacological properties and analytical detectability. Their structural similarity presents challenges in chromatographic separation and mass spectrometric identification.

MDMA (3,4-methylenedioxymethamphetamine) is an amphetamine-type stimulant often used in forensic and clinical toxicology. It is structurally characterized by a methylenedioxy-substituted aromatic ring and possesses both stimulant and serotonergic psychoactive effects (Melo et al., 2024). MDMA, commonly known as ecstasy, is a widely abused psychoactive substance. MDMA is commonly categorized as a psychedelic agent. It exhibits strong empathogenic effects, generating sensations of intense euphoria while simultaneously promoting relaxation and calmness. The compound enhances users’ sense of emotional closeness and reduces anxiety, particularly fears related to rejection or social interaction. People are often drawn to MDMA for its ability to foster interpersonal connection, lower stress levels, boost self-confidence, and enhance sexual experience and performance. Its use is especially prevalent in nightclubs and rave environments, where individuals seek increased energy and sociability throughout extended events (Sottile et al., 2023). MDMA tablets or powders are usually referred to as ““Ecstasy”“ or ““Molly”“. The word ““Ecstasy”“ refers to the tablet form of the drug, whereas ““Molly”“ refers to the crystalline or powder form, which is regarded more pure. ““Ecstasy”“ is often a little, spherical tablet available in a range of colors (Krotulski et al., 2018; Sottile et al., 2023). Analyzing abused substances like MDMA is crucial, as their widespread use has evolved into a global sociopolitical issue affecting populations worldwide. According to 2016 reports by the United Nations (UN) and the United Nations Office on Drugs and Crime (UNODC), around 30 million individuals between the ages of 15 and 64 approximately 0.3% of the global population had used ecstasy. In the same year, Brazil reported the seizure of 133 kg of MDMA, accounting for 0.3% of total global MDMA consumption (Crime Unooda, 2016). In 2021, more than 75% of ecstasy users in the European Union were aged 15–34 years (EMCDDA. European monitoring Centre for Drugs and Drug Addiction, 2007). In a 2019 research of 35 European nations, it was shown that over 100,000 high school students and 2.3% of young people aged 15–16 have taken ecstasy, making it the second most popular drug among this demographic (Molinaro et al., 2019). This raises issues and is a public health problem to address MDMA use is associated with significant toxicological risks, including neurotoxicity, hyperthermia, and cardiovascular complications. The widespread misuse of MDMA and its presence in illicit drug markets highlights the importance of reliable analytical detection methods. In addition to MDMA, structurally similar compounds such as 3,4-methylenedioxyamphetamine (MDA) and 3,4-methylenedioxyethylamphetamine (MDEA) are commonly used in forensic investigations (Figure 5). These compounds have a similar methylenedioxy-substituted aromatic ring, but their side-chain substitutions vary, influencing their pharmacological actions and metabolic routes. The detection of MDMA and its analogs is influenced by their physicochemical properties, metabolic pathways, and structural similarity to other amphetamine-type stimulants. These aspects, along with appropriate analytical platforms and validation considerations, are examined in Section 3.

### 2.6 Methamphetamine (MA)

**FIGURE 6:**
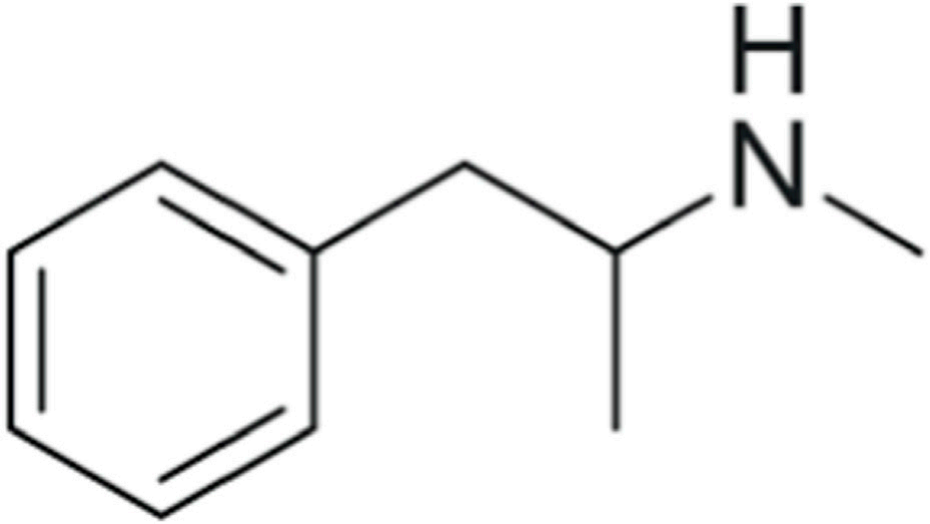
Methamphetamine.

Methamphetamine (meth) (2 S)-N-methyl-1- phenylpropan-2-amine misuse, is one of the fatal health crises that have spread over the world (Figure 6). MA is a synthetic psychoactive drug that belongs to the ATS class, alongside amphetamine and MDMA (3,4 methylenedioxymethamphetamine, ecstasy) (Pérez-Pereira et al., 2024). It is commonly sold under various street names, including crystal meth, chalk, crank, and ice. Crystal meth, also known as shabu in Egypt, is considered one of the most potent forms of the drug (Hashisha et al., 2022), (Kim et al., 2023). It comes in two forms: base, a colorless volatile oil, and hydrochloride salt, a crystalline solid (Manchester et al., 2018). It is classed as a globally regulated substance and may be used both legally and illegally. The FDA (Food and Drug Administration) has authorized it as a second-line therapy for attention-deficit and hyperactivity disorder (ADHD) (Abbruscato et al., 2018) as well as for short-term obesity treatment. MA ranks as the second most commonly abused illicit substance globally, contributing to significant social and public health challenges. Its use is frequently associated with cognitive deficits and the onset of psychotic symptoms that closely resemble those seen in schizophrenia. Moreover, repeated or excessive consumption of MA can result in neurotoxic effects, along with an increased risk of cerebrovascular incidents and even sudden death (Brookfield et al., 2021; Busceti et al., 2021; Mizuno et al., 2021; Sun et al., 2020; Wei et al., 2021). MA is a compound that mainly functions by persistently stimulating nerve cells within the nervous system (Macur and Ciborowski, 2021). Regardless of whether it is taken as a pill or in powder form, this substance causes rapid changes in mood and activates multiple regions of the brain (Abbruscato et al., 2018). According to a research study conducted on the U.S. population, MA use was reported by 0.9% of individuals aged 12 and older (Durell et al., 2008). Furthermore, statistics published by the Centers for Disease Control and Prevention (CDC) reveals an estimated 32,000 fatalities attributable to MA overdose in the year 2021 (Control CfD and Prevention, 2021). Although MA is usually eliminated from the body within 4 days, it can still be identified using blood, urine, or hair analysis (Zokaee et al., 2022). Individuals withdrawing from MA commonly experience a variety of emotional symptoms, such as depression, exhaustion, and increased irritability or aggression (Sanatkar et al., 2022). These withdrawal effects can last for up to 2 weeks, depending on the severity of the addiction, before gradually subsiding. Preventing the development of MA dependence begins with raising public awareness and providing education on its dangers and potential health consequences. The analytical determination of MA is governed by its volatility, basicity, and matrix-dependent distribution, which influence both chromatographic behavior and sensor response. These factors are systematically addressed within the analytical strategies presented in Section 3.

Collectively, the diverse chemical structures, physicochemical properties, and metabolic behaviors of these drug classes highlight the need for carefully tailored analytical strategies. The following section provides a comprehensive evaluation of the analytical platforms used for drug detection, emphasizing the relationship between analyte properties, sample preparation, and method performance.

## 3 General analytical platforms for drug detection

The detection of drugs of abuse relies on a diverse spectrum of analytical platforms, each selected based on the physicochemical characteristics of the target analytes, the complexity of the sample matrix, and the required analytical performance. Rather than representing interchangeable tools, these techniques form a complementary framework in which method selection is dictated by fundamental chemical principles and practical constraints. Broadly, analytical strategies can be categorized into (i) laboratory-based hyphenated techniques, primarily chromatography coupled with mass spectrometry, and (ii) emerging portable systems, including electrochemical, optical, and biosensor-based approaches. While chromatographic–mass spectrometric methods remain the gold standard for confirmatory analysis due to their unmatched sensitivity and specificity, recent advances in sensor technologies have enabled rapid, cost-effective alternatives for on-site screening. A critical understanding of how analyte properties influence extraction, separation, and detection is therefore essential for rational method selection.

### 3.1 Influence of physicochemical properties on method selection

The choice of analytical method is fundamentally governed by the physicochemical properties of drugs and their metabolites, which determine their behavior throughout the analytical workflow. It is important to note that compounds encountered within similar forensic contexts may differ significantly in chemical structure; for example, NBOMe and NBOH compounds are structurally distinct synthetic phenethylamines rather than LSD analogs, despite exhibiting similar pharmacological effects. Parameters such as polarity, molecular weight, volatility, lipophilicity (logP), acid–base characteristics (pKa), and chemical stability directly influence extraction efficiency, chromatographic retention, and detection sensitivity. Structural isomerism represents an additional analytical challenge, particularly for synthetic cathinones, where positional isomers may exhibit very similar physicochemical properties. This similarity can significantly affect chromatographic behavior and complicate reliable compound identification. Highly polar and thermally labile compounds, including many amphetamine-type stimulants and their metabolites, are most effectively analyzed using liquid chromatography–tandem mass spectrometry (LC–MS/MS), which allows direct analysis without derivatization and provides excellent sensitivity at trace levels (Alvarez et al., 2017; Cho et al., 2020; Dolder et al., 2018). In contrast, gas chromatography–mass spectrometry (GC–MS) is better suited for volatile and thermally stable analytes, or compounds that can be derivatized to improve volatility and chromatographic performance, as demonstrated for cathinones and amphetamine derivatives (Abiedalla et al., 2017; Alves et al., 2021; Antunes et al., 2021).

The ionization efficiency of analytes in LC–MS is strongly dependent on their pKa and polarity, which govern their response under electrospray ionization (ESI) conditions and ultimately influence LOD limits and quantitative accuracy (Alvarez et al., 2017; Dolder et al., 2018). Lipophilicity plays a crucial role in matrix distribution and extraction behavior; for example, lipophilic compounds such as SCs exhibit preferential accumulation in hair and fatty tissues, enabling long-term detection (Cho et al., 2020). Chemical stability is a critical factor in analytical method selection, particularly for labile compounds such as psilocin and LSD, which undergo rapid degradation in biological matrices and therefore require prompt extraction and carefully optimized analytical conditions (Cardoso et al., 2023; Zhou et al., 2021). In the case of LSD, its major metabolite, 2-oxo-3-hydroxy-LSD (O-H-LSD), is typically present at higher concentrations in urine than the parent compound, making it a more reliable biomarker for analytical detection (Dos Santos et al., 2025; Jang et al., 2015). These physicochemical considerations also extend to sensor-based platforms, where analyte surface interactions such as hydrophobic, electrostatic, and π–π interactions determine binding affinity and signal response (Duan et al., 2024; Lima et al., 2020; Ribeiro et al., 2020). Consequently, no single analytical method is universally applicable; instead, method selection must be rationally tailored to the chemical nature of the analyte and the analytical context. The relationship between key physicochemical properties and the selection of appropriate analytical techniques is summarized in Table 2.

**TABLE 2:** Relationship between physicochemical properties and analytical method selection.

Property	Analytical implication	Preferred techniques	Example applications
High polarity	Poor volatility	LC–MS/MS	Amphetamines
Low polarity (lipophilic)	Strong matrix retention	LC–MS/MS, hair analysis	SCs
Volatility	Suitable for gas phase	GC–MS	MA
Thermal stability	Required for GC	GC–MS	Cocaine
Low thermal stability	Degrades in GC	LC–MS/MS	Cathinones
Ionizable (pKa)	Affects ionization	LC–MS/MS	Basic drugs
High molecular weight	Not GC-compatible	LC–MS/MS, HRMS	NPS
Chemical instability	Requires rapid prep	LC–MS/MS	Psilocin

### 3.2 Sample pretreatment strategies

Sample pretreatment represents a critical step in the analytical process, often determining the overall sensitivity, selectivity, and reproducibility of the method. Biological matrices such as blood, urine, hair, and OF contain complex mixtures of proteins, lipids, salts, and endogenous metabolites that can interfere with analyte detection, necessitating effective extraction and cleanup procedures. For SCs, particularly in urine, metabolite-based analysis is often essential, as parent compounds are extensively metabolized and may be present at very low concentrations. Targeting metabolites therefore improves detection sensitivity and reduces the likelihood of false-negative results. A comparative overview of commonly used sample pretreatment strategies, including their advantages and limitations, is presented in Table 3. Common sample preparation techniques include protein precipitation (PP), liquid–liquid extraction (LLE), solid-phase extraction (SPE), and microextraction methods. Protein precipitation is widely used for its simplicity and speed, particularly in high-throughput workflows; however, it provides limited selectivity and may not sufficiently remove matrix interferences. Liquid–liquid extraction is effective for nonpolar and moderately polar compounds, exploiting differences in solubility between immiscible phases, but often involves significant solvent consumption and limited selectivity. Solid-phase extraction is considered the most versatile and selective approach, enabling efficient cleanup and preconcentration of analytes from complex matrices. It is particularly valuable in forensic toxicology, where high sensitivity and low background interference are required. More recently, microextraction techniques such as solid phase microextraction (SPME) and dispersive liquid‐liquid microextraction (DLLME) have gained prominence due to their reduced solvent usage, high enrichment factors, and alignment with green analytical chemistry principles (Poliwoda et al., 2020; Williams et al., 2019). The selection of pretreatment strategy is closely linked to both analyte properties and matrix characteristics. For instance, hair analysis requires extensive washing and digestion steps to remove external contamination and release incorporated analytes, whereas OF analysis must address challenges related to low sample volume and variable composition. Effective sample preparation is essential not only for improving analyte recovery but also for minimizing matrix effects that can compromise detection accuracy, particularly in LC–MS/MS analysis. In the case of SCs, analytical strategies frequently focus on metabolite profiling rather than parent compounds. However, shared metabolic pathways (e.g., hydroxylation and carboxylation) can lead to structurally similar metabolites across different compounds, complicating unambiguous identification and necessitating high-resolution or confirmatory analyses (Castaneto M. et al., 2014).

**TABLE 3:** Common sample pretreatment strategies in drug abuse analysis:

Sample preparation technique	Principle	Major advantages	Key limitations	Typical matrices	Commonly detected drug classes
Protein precipitation (PP)	Removal of proteins using organic solvents (e.g., acetonitrile, methanol)	Simple, rapid, low cost, suitable for high-throughput analysis	Limited cleanup efficiency; residual matrix effects	Plasma, serum, whole blood	Amphetamines, MDMA, methamphetamine
Liquid–Liquid extraction (LLE)	Partitioning analytes between aqueous and organic phases	Good recovery for moderately nonpolar compounds; inexpensive	Large solvent consumption; emulsion formation; labor-intensive	Blood, urine, oral fluid	Methamphetamine, MDMA, LSD
Solid-phase extraction (SPE)	Retention of analytes on sorbent followed by selective elution	Excellent cleanup, high selectivity, enhanced sensitivity	Higher cost; multiple processing steps	Blood, urine, oral fluid, hair extracts	Synthetic cannabinoids, cathinones, LSD
solid phase microextraction (SPME)	Adsorption of analytes onto coated fibers	Solvent-free, environmentally friendly, compatible with GC-MS	Limited extraction capacity; fiber fragility	Urine, saliva, seized materials	Volatile and semi-volatile drugs
Dispersive liquid‐liquid microextraction (DLLME)	Fine dispersion of extraction solvent to maximize contact area	High enrichment factors, low solvent consumption	Requires optimization for each analyte/matrix	Urine, environmental samples	Trace-level NPS, cathinones
Hair digestion/Decontamination	Washing and digestion of hair matrix prior to extraction	Enables long-term exposure assessment	Time-consuming; risk of external contamination	Hair	Synthetic cannabinoids, LSD, MDMA
QuEChERS-based extraction	Salting-out extraction followed by dispersive cleanup	Rapid, efficient, reduced solvent use	Limited suitability for highly polar compounds	Seized materials, biological samples	Cathinones, synthetic cannabinoids
Online-SPE/Automated extraction	Automated sample cleanup integrated with LC-MS/MS	High throughput, reduced manual error	Expensive instrumentation	Clinical and forensic laboratories	Multi-drug screening panels

### 3.3 Chromatographic and mass spectrometric techniques

**FIGURE 7:**
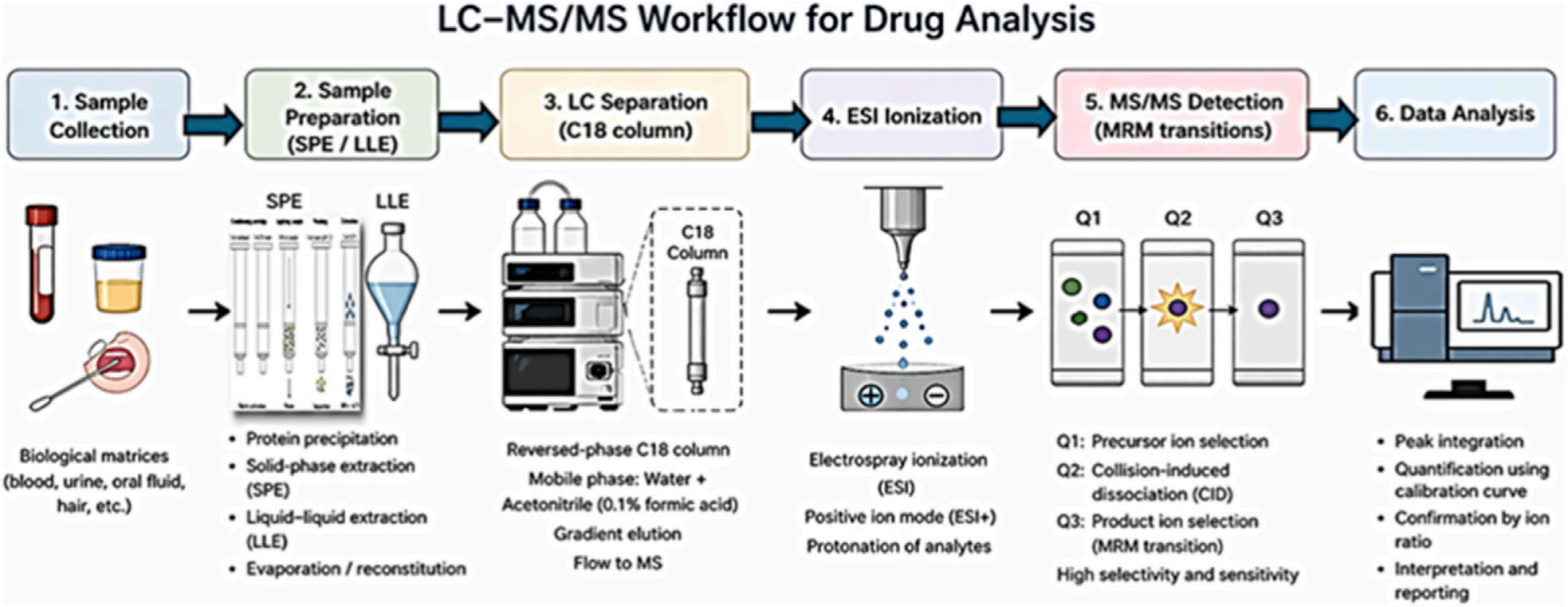
General LC–MS/MS workflow for the detection of drugs of abuse in biological matrices. The process includes sample preparation (e.g., SPE or LLE), chromatographic separation on a reversed-phase C18 column (ESI), tandem mass spectrometric detection using (MRM), and quantitative data analysis.

Chromatographic techniques coupled with mass spectrometry, particularly LC–MS/MS and GC–MS, remain the cornerstone of modern drug detection due to their exceptional sensitivity, selectivity, and multi-analyte capability (Alvarez et al., 2017; Dolder et al., 2018; Faro et al., 2024; Fysh et al., 1985; Williams et al., 2019). These methods are widely regarded as the gold standard for confirmatory analysis in forensic and clinical toxicology. LC–MS/MS is especially suited for the analysis of polar, non-volatile, and thermally labile compounds Figure 7. Typical methods employ reversed-phase columns (most commonly C18 or phenyl-hexyl stationary phases) with gradient elution using aqueous mobile phases (e.g., water with 0.1% formic acid or ammonium formate buffer) combined with organic modifiers such as acetonitrile or methanol. Detection is typically achieved using electrospray ionization (ESI), most often in positive ion mode for basic drugs, coupled with triple quadrupole mass analyzers for highly sensitive and selective quantification. High-resolution mass spectrometry systems, such as quadrupole time-of-flight (QTOF) or Orbitrap instruments, are increasingly used for untargeted screening and retrospective analysis of new psychoactive substances (Alvarez et al., 2017; Cho et al., 2020; Dolder et al., 2018). GC–MS remains indispensable for volatile compounds and analytes amenable to derivatization. Its robustness, reproducibility, and extensive spectral libraries facilitate reliable compound identification and structural elucidation, particularly in forensic casework (Abiedalla et al., 2017; Alves et al., 2021; Antunes et al., 2021). A major challenge in the analysis of synthetic cathinones is the presence of positional isomers, which often produce nearly identical mass spectra and fragmentation patterns. Consequently, accurate identification frequently requires optimized chromatographic separation and, in some cases, the use of complementary techniques such as high-resolution mass spectrometry or spectroscopic methods to achieve reliable discrimination. Additionally, GC-based techniques are valuable for resolving structural isomers when combined with complementary approaches such as infrared spectroscopy. Despite their analytical superiority, chromatographic–mass spectrometric methods are associated with several limitations, including high instrumentation cost, complex operation, and the need for skilled personnel. Furthermore, these techniques are inherently laboratory-bound and often involve time-intensive sample preparation. Nevertheless, their unmatched sensitivity, specificity, and legal defensibility ensure their continued dominance in confirmatory drug analysis.

### 3.4 Electrochemical and sensor-based approaches

**FIGURE 8:**
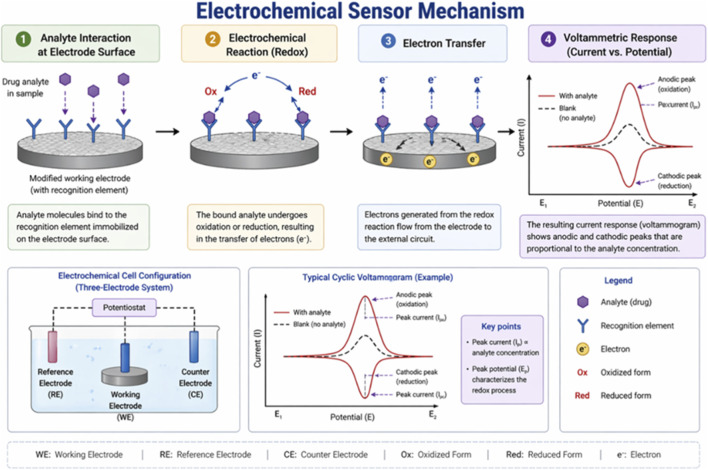
Schematic illustration of the electrochemical sensor mechanism for the detection of drugs of abuse.

Electrochemical and sensor-based platforms have emerged as powerful complementary tools for the rapid detection of drugs of abuse, particularly in field and point-of-care settings. These systems offer significant advantages, including portability, low cost, rapid response, and minimal sample preparation, making them highly suitable for on-site screening applications (Figure 8). Recent advances have focused on enhancing sensor performance through the incorporation of nanomaterials such as carbon nanotubes, graphene, carbon nanofibers, and metal nanoparticles, which improve electron transfer kinetics, increase active surface area, and enhance sensitivity (Duan et al., 2024; Haghighi et al., 2020; Lima et al., 2020; Ribeiro et al., 2020). However, beyond sensitivity, the analytical performance of sensors must be evaluated using a broader set of parameters. Selectivity is a major challenge, especially when detecting structurally similar compounds or analyzing complex matrices. To improve selectivity, various recognition strategies have been developed, including molecularly imprinted polymers (MIPs), aptamer-based sensors, and antibody-functionalized electrodes. These approaches enable specific binding interactions that reduce cross-reactivity and enhance analytical specificity (Duan et al., 2024; Soni et al., 2022).

Matrix effects are particularly significant in biological samples, where endogenous substances such as proteins, salts, and metabolites can interfere with the electrochemical response. Unlike chromatographic techniques, which benefit from separation steps, sensors often operate with minimal pretreatment, making them more susceptible to interference. Strategies such as antifouling coatings, selective membranes, and surface modifications have been employed to improve matrix tolerance. Operational stability and sensor lifetime are critical for practical deployment. Sensor performance can deteriorate over time due to electrode fouling or degradation of recognition elements, leading to signal drift and reduced sensitivity. The development of robust nanocomposite materials and regeneration protocols is therefore essential to enhance durability. Reproducibility and repeatability are also key considerations, particularly for field applications. Variability in electrode fabrication and measurement conditions can affect signal consistency, highlighting the need for standardized preparation and calibration procedures. In addition, sensor-based systems offer rapid response times, often providing results within seconds, which is a significant advantage over chromatographic methods. However, they generally exhibit lower sensitivity and limited quantitative accuracy compared to mass spectrometry, particularly for trace-level detection in complex matrices. As a result, they are primarily used for preliminary screening, with confirmatory analysis performed using laboratory-based techniques. Electrochemiluminescence (ECL), fluorescence, and colorimetric sensors further expand the capabilities of sensor-based detection, offering high sensitivity and simple signal readouts. Despite ongoing challenges related to selectivity and stability, continuous advancements in nanotechnology and surface chemistry are progressively enhancing the analytical performance of these systems. The analytical performance characteristics of the major sensor-based platforms are summarized in Table 4.

**TABLE 4:** Comparative performance of sensor-based methods.

Method type	Sensitivity (LOD)	Selectivity	Stability/Lifetime	Reproducibility	Matrix tolerance	Key advantages	Limitations
Electrochemical sensors	Low (nM–µM)	Moderate–High (with modification)	Moderate (fouling issues)	Moderate	Limited	Fast, portable, low cost	Matrix interference
MIP-based sensors	Low	High (specific cavities)	Good	Good	Moderate	High selectivity	Fabrication complexity
Aptamer-based sensors	Very low (nM–pM)	Very high	Moderate	Good	Moderate	Excellent specificity	Stability issues
ECL sensors	Very low	High	Good	Good	Moderate	High sensitivity, low noise	Instrumentation needed
Fluorescence sensors	Low–very low	Moderate–high	Moderate	Good	Limited	Simple, rapid detection	Interference from matrix
Colorimetric sensors	Moderate	Low–moderate	High	Good	Low	Visual detection, easy use	Low sensitivity
surface-enhanced Raman scattering (SERS)-based sensors	Very low	High	Good	Moderate	Moderate	Molecular fingerprinting	Reproducibility challenges

### 3.5 Optical and spectroscopic methods

Optical and spectroscopic techniques, including fluorescence sensing, Raman spectroscopy, SERS, and colorimetric assays, have gained increasing attention due to their rapid response, simplicity, and potential for field deployment (Braz et al., 2021; Kumar et al., 2023; Yen et al., 2019; Yen et al., 2022). These methods rely on analyte-induced changes in optical properties, enabling qualitative or semi-quantitative detection. Fluorescence-based sensors utilize molecular probes or nanomaterials that interact selectively with target analytes, producing measurable changes in emission intensity. Raman and SERS techniques provide molecular fingerprint information, enabling highly specific identification even in complex mixtures. Colorimetric assays allow visual detection without sophisticated instrumentation, making them particularly suitable for rapid screening. However, these techniques face challenges related to selectivity, signal variability, and matrix interference. Environmental factors and overlapping spectral features can affect reproducibility and quantitative reliability. Advances in chemometric analysis and machine learning are increasingly being used to improve signal interpretation and enhance analytical performance. A direct comparison of representative chromatographic and sensor-based methods for selected drugs of abuse is presented in Table 5.

**TABLE 5:** Comparative analytical performance of chromatographic and sensor-based methods for drug detection.

Drug	Method	Matrix	LOD	LOQ	Accuracy (%)	Recovery (%)	Key notes
LSD	LC–MS/MS	Plasma	0.01 ng/mL	0.05 ng/mL	95–105	85–110	Gold standard, high sensitivity
LSD	Electrochemical sensor	Blotter	0.38 μmol/L	—	∼90	∼80	Rapid, but less sensitive
Cathinones	LC–MS/MS	Urine	<1 ng/mL	2–5 ng/mL	92–108	80–105	Multi-analyte capability
Cathinones	Raman/SERS	Seized samples	∼0.4 ppm	—	∼85	—	Field screening
SCs	LC–MS/MS	OF	0.1–1 ng/mL	1–5 ng/mL	90–110	80–100	High selectivity
SCs	Electrochemical sensor	Herbal	∼µM range	—	∼85	—	Portable, limited specificity
MDMA	LC–MS/MS	Plasma/urine	0.1 ng/mL	0.5 ng/mL	95–105	85–110	Confirmatory method
MDMA	Electrochemical	OF/tablets	0.6 μmol/L	—	∼90	∼85	Fast screening
MA	GC–MS	Blood/urine	0.05–0.5 ng/mL	1 ng/mL	92–108	80–110	Highly reliable
MA	Colorimetric sensor	Field samples	0.36 μg/mL	—	∼80	—	Simple, low sensitivity

### 3.6 Integrated analytical strategies

The growing complexity of illegal drug markets, particularly the fast introduction of new psychoactive substances (NPS), has shown the difficulties of using a single analytical platform for all forensic and therapeutic applications. Instead, modern drug detection is increasingly using a two-tiered analytical approach that combines quick screening approaches with highly selective confirmation procedures. This integrated process aims to strike a compromise between speed, affordability, mobility, and analytical dependability. The applicability of different analytical platforms across major drug classes is summarized in Table 6, highlighting the complementary strengths and limitations of laboratory-based and field-deployable approaches.

**TABLE 6:** Summary of advanced analytical techniques for selected drugs of abuse.

Analytical approach	Typical techniques	Primary purpose	Sensitivity	Selectivity	Portability	Turnaround time	Main limitations	Typical applications
Initial screening	Immunoassays	Rapid presumptive testing	Moderate	Moderate	High	Minutes	Cross-reactivity; false positives	Workplace testing, emergency departments
Initial screening	Colorimetric assays	Visual detection of target drugs	Moderate	Low	Very high	Seconds–minutes	Poor specificity	Roadside testing, seized materials
Initial screening	Electrochemical sensors	Rapid field detection	Moderate–High	Moderate–High	High	Seconds	Electrode fouling; matrix effects	Point-of-care and forensic screening
Initial screening	Raman/SERS spectroscopy	Molecular fingerprinting	High	High	High	Minutes	Substrate variability; fluorescence interference	Customs, border control, field analysis
Initial screening	Fluorescence sensors	Rapid trace detection	High	Moderate–High	Moderate	Minutes	Matrix interference	Biological sample screening
Confirmatory analysis	GC–MS	Definitive identification and quantification	Very high	Very high	Low	Hours	Requires derivatization for some analytes	Forensic toxicology laboratories
Confirmatory analysis	LC–MS/MS	Gold-standard targeted analysis	Very high	Very high	Low	Hours	Expensive instrumentation	Clinical and forensic confirmation
Confirmatory analysis	LC–HRMS/QTOF	Untargeted screening and retrospective analysis	Very high	Very high	Low	Hours	Complex data processing	NPS detection and metabolite profiling
Hybrid strategy	Sensor + LC–MS/MS	Screening followed by confirmation	Very high	Very high	Moderate	Minutes to hours	Requires multi-platform workflow	Modern forensic drug testing
Hybrid strategy	HRMS + targeted LC–MS/MS	Untargeted discovery followed by quantification	Very high	Very high	Low	Hours	Cost and expertise requirements	Emerging NPS investigations

In the first tier, screening technologies such as immunoassays, electrochemical sensors, colorimetric assays, fluorescence-based platforms, and surface-enhanced Raman scattering (SERS) are used to quickly identify possible drug exposure. These methods often need little sample preparation, have fast processing timeframes, and may frequently be used directly at the point of need, such as roadside testing, emergency clinics, customs inspections, jails, workplaces, and forensic field investigations (Braz et al., 2021; Melo et al., 2025; Rocha et al., 2025). Their primary responsibility is to discover probable positive samples that require additional testing.

The second tier includes laboratory-based confirmatory analysis, such as liquid chromatography-tandem mass spectrometry (LC-MS/MS), gas chromatography-mass spectrometry (GC-MS), and high-resolution mass spectrometry (HRMS) (Fan et al., 2020; Pascual-Caro et al., 2020). These approaches allow unequivocal identification and quantification due to their high sensitivity, selectivity, and legal defensibility. Confirmatory analysis is especially critical when dealing with structurally identical compounds, positional isomers, trace-level analytes, or newly developing NPS, which might provide confusing findings in preliminary screening methods (Faro et al., 2024; Williams et al., 2019).

The practical usefulness of this integrated method is proven across the several medication classes included in this review. Portable electrochemical sensors for LSD allow for the fast testing of confiscated blotter samples, whilst LC-MS/MS and LC-HRMS give final confirmation in biological specimens (Caspar et al., 2018; Dolder et al., 2018). Synthetic cathinones and cannabinoids also benefit from fast field screening followed by chromatographic-mass spectrometric confirmation to address structural diversity and metabolite identification. Electrochemical, colorimetric, and spectroscopic screening methods can identify MDMA and methamphetamine in tablets, saliva, or confiscated materials quickly, although LC-MS/MS and GC-MS are still required for forensic confirmation and toxicological interpretation (Faro et al., 2024; Melo et al., 2024).

Beyond analytical performance, the two-tiered strategy has significant operational advantages. It decreases laboratory burden by restricting confirmatory testing to presumptive positive samples, speeds up decision-making, lowers total analytical costs, and enables large-scale monitoring programs. Furthermore, the growing use of portable sensors, microextraction methods, artificial intelligence-assisted data interpretation, and high-resolution mass spectrometry is likely to improve this process in future forensic and therapeutic practice. As a result, the future of drug detection is unlikely to rely on a single analytical platform. Rather, the most effective strategy will involve the coordinated use of rapid screening technologies and highly selective confirmatory methods, resulting in a comprehensive framework capable of addressing both routine toxicological investigations and the evolving challenges posed by new psychoactive substances. A quantitative comparison of analytical performance across different techniques is summarized in Tables 7, 8, highlighting the significant sensitivity gap between chromatographic and sensor-based approaches.

**TABLE 7:** General comparison of analytical techniques for drug detection.

Technique	Typical LOD	Accuracy	Recovery	Advantages	Limitations
LC–MS/MS	pg–ng/mL	High (95%–105%)	80%–110%	High sensitivity, multi-analyte	Expensive, lab-based
GC–MS	ng/mL	High (90%–105%)	80%–110%	Robust, library matching	Requires derivatization
LC–HRMS	pg–ng/mL	High	80%–100%	Untargeted screening	Complex data analysis
Electrochemical sensors	µM–nM	Moderate (∼85–95%)	70%–90%	Portable, fast	Matrix interference
Optical (fluorescence/SERS)	nM–µM	Moderate	—	Rapid, visual detection	Selectivity issues
Colorimetric tests	µg/mL	Low (∼70–85%)	—	Simple, cheap	Low sensitivity, false positives

**TABLE 8:** Summary of performance gap between laboratory and field-based detection methods.

Parameter	Chromatographic methods	Sensor-Based methods
Sensitivity	Very high (pg–ng/mL)	Moderate (nM–µM)
Selectivity	Excellent	Moderate
Quantification	Accurate	Limited
Speed	Slow (minutes–hours)	Very fast (seconds)
Portability	No	Yes
Suitability	Confirmatory analysis	Screening

### 3.7 Integrated two-tier analytical strategy: Screening and confirmatory approaches

No single analytical platform can simultaneously satisfy all requirements of drug abuse testing, including rapid turnaround, high sensitivity, broad analyte coverage, portability, and legal defensibility. Consequently, modern forensic and clinical toxicology increasingly relies on a two-tier analytical strategy that combines rapid screening methods with highly selective confirmatory techniques.

In the first tier, rapid screening methods such as immunoassays, electrochemical sensors, colorimetric assays, fluorescence-based platforms, Raman spectroscopy, and surface-enhanced Raman scattering (SERS) provide fast and cost-effective preliminary identification of suspected substances. These approaches are particularly valuable for roadside testing, workplace drug screening, emergency departments, customs inspections, and field investigations where immediate decision-making is required. Their major advantages include portability, minimal sample preparation, low operational costs, and rapid response times. However, they may suffer from matrix interferences, cross-reactivity, and limited specificity, particularly when structurally similar compounds or emerging NPS are present.

The second tier involves confirmatory laboratory-based analysis using LC–MS/MS, GC–MS, or high-resolution mass spectrometry (HRMS). These techniques provide superior selectivity, sensitivity, and quantitative reliability, enabling definitive identification and legal confirmation of drugs and metabolites. Confirmatory methods are essential when analytical results may have forensic, clinical, occupational, or judicial consequences.

The integration of these complementary approaches offers several practical advantages. Rapid screening reduces the analytical burden by prioritizing samples requiring detailed examination, while confirmatory methods minimize false-positive and false-negative results. Furthermore, untargeted HRMS screening combined with targeted LC–MS/MS quantification provides a powerful framework for addressing the growing challenge posed by new psychoactive substances. As portable sensing technologies continue to improve, future analytical workflows are expected to become increasingly integrated, linking field-deployable screening systems with centralized laboratory confirmation to achieve faster, more reliable, and cost-effective drug monitoring.

#### 3.7.1 Some cases study

1. In this case the patient is a 24-year-old boy who has been addicted to phensidyl for 4.5 years. He cannot accomplish anything without it. If he is unable to cope, he may have a peevish demeanor and refuse to work or communicate. He obtains narcotics from certain locations or individuals. He began with ‘ganja’. He adjusts his drug use to get his desired degree of enjoyment. He alternates between ‘ganja’, ‘wine’, and ‘phensidyl’. He has been hooked to ‘phensidyl’ for 4.5 years and needs to take it four times daily (Islam et al., 2012).
2. This study found that there is a perception of drug abuse in secondary schools. While most students have a negative opinion, 31.4% of abusers have a favorable perception. A cognitive restructuring program is recommended. Alcohol, cigarettes, and khat were among the most often misused narcotics due to their availability, legality, and cultural acceptance (Kyalo and Mbugua, 2011).
3. This study focuses to investigate on the current trend of drug and substance abuse trends among secondary school students in Kenya, the approaches the schools (Njoki, 2013).
4. A 36-year-old Portuguese man was hit by a train and died on the railway line. His limbs were partially severed, and he suffered terrible injuries. The cause of his death remains unknown, and no additional identification was discovered outside his clothing. The accident site revealed an unidentified automobile with a key in the ignition. The local authorities successfully identified the owner. The investigation determined that the automobile belonged to the victim. The man’s family reported that he had been missing for over 3 days, with one parent mentioning his drug use (Margalho et al., 2011).

## 4 Practical challenges in drug detection: Insights from real-world case studies

While the analytical methodologies covered in this paper are effective instruments for drug identification, real-world forensic case studies reveal substantial practical issues that are not always adequately addressed in controlled laboratory settings. The combination of sample examples including counterfeit medications, synthetic cannabinoid mixes, polydrug formulations, and cannabis adulteration indicates significant shortcomings in both on-site law enforcement detection and clinical toxicological interpretation.

### 4.1 Challenges in on-site law enforcement detection

One of the most critical obstacles emerges during on-site law enforcement operations, when prompt identification is essential. In many situations, confiscated items are purposefully intended to resemble real products, rendering visual identification ineffective. Counterfeit pharmaceutical pills, for example, might be mistaken for genuine drugs in terms of look and packaging, whilst herbal products containing SCs are frequently sold as natural cannabis. Field deployable screening techniques, such as colorimetric assays and portable electrochemical or optical sensors, are frequently utilized in such situations due to their speed and convenience of use. However, these approaches are intrinsically constrained by low selectivity, sensitivity to cross-reactivity, and the inability to detect undiscovered or developing chemicals. As demonstrated in studies using SCs and NPS, the existence of structurally varied and fast changing analogs frequently leads to false negatives or partial identification. Furthermore, polydrug formulations, which mix many psychoactive drugs into a single product, provide an additional hurdle. In such instances, field testing may only identify one component or fail completely owing to signal interference, exposing the limits of single-target detection methods.

### 4.2 Challenges of clinical diagnosis and toxicological interpretation

Clinical toxicology poses a unique set of issues, especially when there is a discrepancy between predicted and actual physiological consequences. Cases of drug replacement or adulteration can dramatically change a substance’s pharmacological profile, creating diagnostic confusion. For example, counterfeit medications with replaced active ingredients may not elicit the predicted therapeutic or harmful effects, thereby delaying necessary medical action. Similarly, the inclusion of unanticipated adulterants, such as medicinal chemicals in cannabis, might cause aberrant clinical manifestations, complicating diagnosis. Polydrug exposure exacerbates these issues. The combined effects of SCs, opioids, stimulants, and other medications can cause complicated and overlapping symptoms, making it difficult to determine the causal agent purely by clinical observation. This emphasizes the importance of broad toxicological screening rather than specialized testing.

### 4.3 Analytical method limitations highlighted by case studies

#### 4.3.1 Matrix effects and emerging analogues

The cases highlight numerous significant shortcomings of existing analytical methods. Although chromatographic methods like LC-MS/MS and GC-MS are regarded as gold standards due to their high sensitivity and specificity, they are constrained by their reliance on laboratory infrastructure, time-consuming sample preparation, and the need for skilled staff. These limits limit their usefulness in rapid-response circumstances. One significant restriction is the reliance on reference standards and spectrum libraries. Because new psychoactive chemicals are constantly being discovered, many compounds are missing from current databases, making detection challenging even with modern equipment. This is especially troublesome for SCs and cathinones, as structural similarity and isomerism make spectrum interpretation difficult.

In addition, matrix complexity is important. Biological samples and plant-based materials contain a diverse set of interfering chemicals that might impair extraction efficiency, chromatographic separation, and detection sensitivity. In circumstances involving contaminated cannabis or counterfeit medications, complex matrices can obscure the presence of active chemicals or suppress signals. While sensor-based approaches show promise for quick screening, they are nevertheless restricted by selectivity and matrix interference. Although advances in nanomaterials and molecular recognition elements have increased performance, these systems still lack the durability and validation necessary for confirmatory testing.

#### 4.3.2 Certified reference materials (CRM) and quantitative strategies for new psychoactive substances

The rapid emergence and structural diversification of NPS, such as SCs, synthetic cathinones, and nitazene class synthetic opioids, has posed significant analytical challenges due to the scarcity of certified reference materials (CRMs). Accurate identification and quantification in forensic and clinical toxicology are dependent on verified reference standards for calibration, validation, and confirmatory analysis (EMCDDA IJLPOotEU, 2017). However, the rate of illegal market innovation frequently outpaces commercial providers’ capacity to synthesis, describe, and disseminate new CRMs, causing delays between substance identification and standard availability.

Established drugs of abuse have readily available CRMs from certified providers, but newly developing NPS typically lack rapid market requirements. CRM development necessitates structural confirmation, purity assessment, homogeneity testing, and stability evaluation, all of which are time-consuming and regulated operations (EMCDDA IJLPOotEU, 2017). This constraint is especially troublesome for extremely powerful chemicals like nitazenes, where safe handling, regulatory categorization, and synthesis logistics all impede quick distribution (Moody et al., 2018). In the absence of CRMs, labs use alternative quantitative methodologies. One typical strategy is to employ surrogate standards, which are structurally similar counterparts used for temporary calibration (Hutter et al., 2012). Although technique allows for approximate concentration determination, changes in ionization efficiency and matrix effects can induce bias. Similarly, isotopically labeled internal standards from the same structural class can be employed to account for extraction recovery and ion suppression, although they do not entirely compensate for the lack of compound-specific calibration (Hutter et al., 2012).

High-resolution mass spectrometry (LC-HRMS or QTOF-MS) has become a critical technique for overcoming CRM shortages. Accurate mass measurement, isotope pattern recognition, and fragmentation libraries enable robust qualitative identification in the absence of legitimate reference standards (Albertstr and Freiburg, 2016). Additionally, HRMS allows for retroactive data analysis when additional compounds are discovered after initial screening. However, reliable quantitative determination is still dependent on verified calibration standards, which limits HRMS applicability for court-defensible reporting without CRM assistance. Other interim solutions include response factor estimates using structurally related chemicals and temporary standard addition methods. While valuable for intelligence and surveillance, these approaches are not recommended for definite forensic quantification due to variability in analytical reaction (Armenian et al., 2018; Hutter et al., 2012).

Recent research has centered on expediting CRM development by improving coordination among forensic laboratories, early warning networks, and commercial chemical makers. International monitoring methods such as the UNODC SMART Programme and the EMCDDA Early Warning System enable the quick structural detection and distribution of developing NPS information, potentially lowering the time to CRM production (Armenian et al., 2018; Krotulski et al., 2018). Furthermore, developing common spectrum databases improves non-targeted detection capabilities while waiting for recognized standards. Despite these advancements, CRM scarcity remains a substantial barrier to inter-laboratory repeatability, regulatory compliance, and toxicological dependability. To alleviate this analytical bottleneck, regulatory authorities, analytical laboratories, and suppliers must work together more effectively, as well as invest in high-resolution screening technology. Nonetheless, accurate quantitative analysis remains dependent on the availability of verified reference materials, emphasizing CRM access as a significant obstacle in modern NPS detection.

### 4.4 Implications of future analytical strategies

The issues described in these case studies emphasize the need of using integrated, multi-tiered analytical methodologies. No single strategy is enough to address the complexities of current illegal drug compositions. Instead, efficient drug detection necessitates the employment of quick screening techniques for on-site application in conjunction with extremely sensitive and selective laboratory-based confirmation procedures.

Future advancements should concentrate on extending spectrum libraries, adding high resolution mass spectrometry for untargeted screening, and increasing the selectivity and robustness of portable detection devices. Furthermore, the integration of forensic intelligence, data exchange, and early warning systems will be important in dealing with the quickly changing drug addiction scene. Overall, these case studies show that breakthroughs in analytical chemistry must be supported by practical application to real world situations in order to effectively support law enforcement, clinical diagnosis, and public health measures.

## 5 Regional considerations and adaptation of detection technologies

Although the fundamental principles of drug detection are globally applicable, regional differences in drug abuse patterns influence analytical priorities and method selection. In the Middle East and North Africa (MENA) region, including the United Arab Emirates (UAE) and Egypt, the illicit drug landscape differs in several respects from that reported in North America and Europe. In Gulf countries, amphetamine-type stimulants, including methamphetamine and Captagon-related substances, together with synthetic cannabinoids and emerging NPS, represent major concerns for forensic and law-enforcement agencies. Consequently, rapid screening technologies suitable for border control, customs inspections, and field deployment have gained increasing importance. Portable electrochemical sensors, Raman spectroscopy, and handheld spectrometers can provide rapid preliminary identification, while LC–MS/MS and HRMS remain essential for confirmatory analysis. In Egypt and neighboring North African countries, misuse of tramadol, cannabis products, methamphetamine, and synthetic cannabinoids has been widely reported. These substances are frequently encountered in forensic and clinical toxicology laboratories, emphasizing the need for cost-effective, high-throughput analytical workflows. Immunoassay screening combined with LC–MS/MS confirmation represents a practical strategy for routine monitoring, while HRMS offers additional capability for the identification of emerging psychoactive substances. These regional differences illustrate that analytical method selection should be guided not only by analyte properties but also by local patterns of substance abuse, available laboratory infrastructure, regulatory requirements, and public health priorities. Therefore, adaptable analytical frameworks that combine portable screening technologies with centralized confirmatory testing are likely to provide the most effective response to evolving drug trends in different geographic regions.

## 6 Discussion

The analytical techniques mentioned in the preceding sections demonstrate substantial progress in the identification of drugs of abuse across a variety of matrix and chemical classes. However, these methodologies are being challenged by a number of linked issues, including the fast advent of NPS, complex matrix effects, and an increased desire for sustainable analytical practices. These issues are not separate; rather, they have a combined impact on technique development, detection reliability, and the overall efficacy of forensic and clinical toxicology processing. The advent of NPS greatly increases analytical complexity, as newly produced compounds frequently lack reference standards and may share physicochemical features with older medications. This problem is exacerbated by matrix effects, in which biological components interfere with analyte ionization and detection, particularly in methods like LC-MS/MS. As a result, reliable detection of NPS necessitates not only improved instruments, but also efficient sample preparation procedures and strong analytical validation.

This review has synthesized a broad spectrum of analytical techniques for detecting drugs of abuse, from established laboratory gold standards to emerging field-deployable sensors. The overarching narrative is one of a field in dynamic tension, balancing the uncompromising demand for analytical rigor against the growing need for rapid, accessible, and cost-effective solutions. The comparative analysis reveals that the choice of an optimal method is not a one-size-fits-all proposition but is critically dependent on the specific context be it a high-throughput forensic laboratory, a clinical emergency room, or a roadside law enforcement check.

### 6.1 The inherent trade-off: Sensitivity/specificity vs. speed/portability

A central theme emerging from this review is the fundamental trade-off between the performance of laboratory-based techniques and the practicality of portable platforms. Chromatography coupled with mass spectrometry (LC-MS/MS, GC-MS) remains the undisputed gold standard for confirmatory analysis, providing peerless sensitivity exemplified by LODs in the low pg/mg range for hair analysis (Castaneto M. et al., 2014; Sarafraz-Yazdi and Amiri, 2010) and the ability to unequivocally identify and quantify a vast array of analytes and their metabolites simultaneously. This capability is indispensable for definitive legal proof, postmortem toxicology, and advanced pharmacokinetic studies.

While laboratory-based technologies like LC-MS/MS and GC-MS have unrivaled sensitivity and specificity, their dependence on centralized infrastructure restricts their use in time-critical situations like roadside testing or emergency clinical settings. Portable electrochemical and optical sensors, on the other hand, provide fast, on-site detection but frequently lack the sensitivity needed for trace-level detection in complicated biological matrices. This basic trade-off emphasizes that these techniques should not be considered as competing technologies, but rather as complimentary tools in a multi-tiered analytical framework.

However, this analytical power comes at a cost: it is time-consuming, requires expensive instrumentation and highly skilled personnel, and is inherently confined to the laboratory. In contrast, electrochemical and optical biosensors offer a paradigm shift towards rapid, on-site screening. The work on LSD detection, for instance, illustrates this dichotomy: while LC-MS/MS can quantify LSD in plasma at 0.01 ng/mL (Alvarez et al., 2017), electrochemical sensors for blotter papers operate in the µM range (0.05–0.69 μmol/L) (Dos Santos et al., 2025; Ribeiro et al., 2020). For many field applications, this lower sensitivity may be sufficient for a simple ““detect/no-detect”“ outcome, but it currently precludes their use for quantifying trace levels in complex biological matrices like blood or urine. Furthermore, while sensor selectivity is continually improving through molecular imprinting (90, 126) and aptamer engineering (119), these platforms remain more vulnerable to matrix interferences and cross-reactivity than mass spectrometric detection, potentially leading to false positives in unconfirmed screenings.

Despite tremendous progress, several difficulties persist. Portable sensors are frequently restricted by matrix effects, calibration instability, and decreased selectivity in the presence of structurally related substances. In contrast, laboratory-based procedures have limits in terms of cost, turnaround time, and the requirement for highly skilled staff. To bridge this gap, more hybrid systems must be developed, sensor validation processes enhanced, and advanced data analysis technologies such as machine learning integrated. The comparative analysis of laboratory-based and field-deployable techniques reveals distinct strategic profiles for each approach.

### 6.2 The persistent challenge of NPS

The continuous emergence of NPS represents perhaps the most significant challenge to contemporary forensic toxicology. The structural agility of SCs, cathinones, and hallucinogenic analogs creates a moving target that routinely outpaces the development of reference standards and the updating of targeted analytical methods. This review highlights the critical importance of untargeted screening approaches using (LC-HRMS, QTOF) (61, 77, 97) in this context. These platforms allow for retrospective data analysis, enabling the identification of novel compounds after they have been initially detected, even if they were not originally targeted.

The case of LSD analogs like 1P-LSD is particularly illustrative of the interpretive challenges posed by NPS. The detection of only LSD and not the parent 1P-LSD in biological samples (Antunes et al., 2021) suggests *in vivo* conversion, raising a critical forensic question: did the individual consume LSD itself or a legal prodrug? This ambiguity has profound implications for legal proceedings and underscores that analytical findings cannot be interpreted in a vacuum; they require integrated toxicological knowledge.

### 6.3 Matrix selection: Beyond the traditional blood and urine

The expansion into alternative matrices like hair, OF, and sweat is a key development, yet it introduces its own set of complexities. While hair analysis provides an unparalleled historical record of drug exposure (Libanio Osorio Marta RFJDmr, 2019), the interpretation of quantitative results is fraught with challenges. Factors such as hair color, cosmetic treatments, and variability in growth rates can significantly impact drug incorporation, making it difficult to correlate concentration with the timing, frequency, or dose of use. Similarly, OF offers a non-invasive window to recent intake, but drug concentrations can be influenced by collection method (stimulated vs. unstimulated), pH, and the recovery efficiency from the collection device (Samyn and CJIjolm, 2000). While OF offers a non-invasive window to recent intake, it is important to note that OF drug concentrations do not always show a strong correlation with corresponding blood levels, complicating the estimation of systemic impairment. The instability of certain analytes, such as psilocin in OF (99), further complicates reliable quantification. The absence of universally accepted cutoff concentrations and interpretive guidelines for these matrices, unlike the well-established standards for urine, remains a significant barrier to their standardized application in legal and workplace settings.

### 6.4 The critical need for standardization and harmonization

A critical gap identified across the reviewed literature is the lack of standardization in analytical protocols. Variability in sample preparation (e.g., SPE vs. LLE), calibration methodologies, and validation parameters across different laboratories hinders the direct comparability of results and undermines the consistency of forensic science. Adherence to international validation guidelines (e.g., SWGTOX, FDA) is not yet universal. Furthermore, the field would greatly benefit from the wider availability of certified reference materials for NPS and their major metabolites, which are essential for method validation and quality control.

### 6.5 The emergence of green analytical chemistry and sustainability

As the volume of toxicological testing grows, the environmental impact of analytical methods can no longer be ignored. Traditional techniques often rely on large volumes of hazardous organic solvents. In parallel, the growing emphasis on green analytical chemistry necessitates extra concerns in technique development. Efforts to reduce solvent use, waste, and energy efficiency must be balanced with the necessity for excellent sensitivity and selectivity, especially when assessing trace-level NPS in complex biological matrices. As a result, the development of long-term analytical methodologies must keep pace with the changing problems given by NPS detection and matrix-related interferences.

The evaluation of MDMA detection methods using greenness assessment tools (123) is a welcome development that should be adopted more broadly. The trend towards miniaturization, seen in micro-extraction techniques (Reyes-Garces et al., 2017) and portable sensors, aligns perfectly with the principles of Green Analytical Chemistry (GAC). Future method development should explicitly prioritize the reduction of waste, energy consumption, and the use of hazardous substances without compromising analytical performance.

Adopting microextraction methods has shown to be one of the most effective GAC strategies. Solid phase microextraction (SPME) removes or significantly decreases organic solvent use by extracting analytes directly onto coated fiber (De Coning and Stølsvik, 2013; Pawliszyn, 2011). In forensic drug analysis, SPME has been effectively used to determine amphetamine-type stimulants, MA, and volatile drug residues in biological matrices, resulting in significant enrichment factors while decreasing sample handling and waste formation (Reyes-Garces et al., 2017). Similarly, dispersive liquid-liquid microextraction (DLLME) uses microliters of extraction solvent and has shown remarkable enrichment potential for synthetic cathinones, MDMA, and other novel psychoactive compounds in urine and OF. DLLME generally consumes more than 90% less solvent than traditional liquid-liquid extraction while retaining comparable recoveries and LOD (Sarafraz-Yazdi and Amiri, 2010). Pretreatment techniques that are solvent-free and downsized help to promote more sustainable analytical operations. The automated single-drop microextraction-capillary electrophoresis (SDME-CE) method published for psilocin and muscimol detection in urine is an instructive example, reaching a 170-fold enrichment factor with low reagent use and high sensitivity. Such methods greatly reduce solvent consumption while still providing analytical performance adequate for forensic toxicology applications.

Another key part of GAC is the substitution of hazardous chemicals with less harmful replacements. In place of chlorinated solvents and very toxic extraction reagents, modern drug detection techniques increasingly use aqueous mobile phases, ethanol-based extraction systems, biodegradable polymeric sorbents, and environmentally friendly buffers. Furthermore, electrophoretic methods and portable electrochemical sensors use far less reagent volume than traditional chromatographic procedures, lowering environmental impact. The increasing usage of paper-based analytical equipment and 3D-printed electrochemical platforms for MDMA, LSD, and synthetic cannabinoid screening helps to minimize material consumption and simplify waste management. The environmental performance of analytical procedures may be objectively evaluated using greenness criteria. Furio-Sanz et al. assessed twenty published MDMA analytical techniques using four complementing assessment tools: Analytical GREEnness (AGREE), Analytical Eco-Scale (AES), Green Analytical Procedure Index (GAPI), and the National Environmental Methods Index (NEMI). Their findings showed that electrophoretic techniques, downsized extraction processes, and green solvent-based technologies consistently outperformed traditional solvent-intensive chromatographic protocols in terms of greenness. The authors also advocated for the combined use of different assessment methodologies to give a more thorough examination of analytical sustainability.

Overall, future analytical techniques for drugs of abuse should embrace GAC concepts from the outset of method development. Microextraction techniques, solvent-free sample preparation, low-toxicity reagents, miniaturized analytical platforms, and formal greenness assessment tools must all be integrated to achieve environmentally sustainable forensic and clinical toxicology while maintaining trace-level drug detection sensitivity and selectivity.

### 6.6 Synthesis and integration: The path forward

No single analytical platform can address all the demands of modern drug detection. The future lies not in the supremacy of one technique over another, but in their strategic integration. The most robust framework involves a synergistic two-tiered system: first, rapid on-site screening using highly portable and cost-effective electrochemical or colorimetric sensors (43, 117) to provide immediate intelligence for law enforcement or clinical triage; followed by confirmatory analysis in a centralized laboratory using LC-MS/MS or GC-MS for definitive, court-admissible results. Advances in data analytics, including machine learning for SERS data interpretation (130) and chemometric analysis for sensor optimization (Albertstr and Freiburg, 2016), will further enhance the reliability and discriminatory power of both laboratory and field-based methods. By embracing this complementary approach, the field can evolve to more effectively combat the dual challenges of traditional drug abuse and the relentless proliferation of NPS.

These problems underscore the necessity for integrated analytical methodologies that include high-resolution instruments, sophisticated sample preparation techniques, and long-term methodological design. Developing strong, future-ready analytical procedures in forensic and clinical toxicology requires addressing NPS variety, matrix effects, and environmental issues all at the same time.

## 7 Future prospects

Analytical chemistry, artificial intelligence, sophisticated materials science, and digital health technologies are predicted to come together to determine the future of drug misuse detection. As illegal drug markets change, particularly with the fast advent of new psychoactive substances (NPS), future analytical systems must offer high sensitivity, broad analyte coverage, mobility, quick turnaround, and environmental sustainability.

### 7.1 Smartphone-integrated and portable detection systems

One of the most exciting advances is the incorporation of analytical sensors into cellphones and wireless communication systems. Smartphone-assisted electrochemical, fluorescence, and colorimetric sensors have previously proven the potential to collect real-time data, interpret signals, and transmit results utilizing portable devices. Future systems are projected to merge tiny sensing platforms with cloud-based databases, allowing for quick drug detection in the field via automated comparison to centralized spectrum libraries. Such technologies have the potential to greatly enhance drug screening in several situations, including roadside testing, customs inspections, emergency departments, prisons, and distant treatment sites.

### 7.2 Artificial intelligence and machine learning-assisted analysis

AI and ML are rapidly being used in analytical procedures. Advanced algorithms can handle complicated chromatographic, spectroscopic, and electrochemical information, allowing for automatic pattern identification, compound categorization, and better interpretation of overlapping signals. AI-assisted analysis of Raman spectroscopy, surface-enhanced Raman scattering (SERS), and high-resolution mass spectrometry (HRMS) data, in particular, has the potential to speed up the detection of new psychoactive drugs before certified reference materials are available. Future forensic laboratories are likely to use integrated AI systems that can constantly update spectral databases and predict the identity of newly emerging compounds.

### 7.3 Dried blood spot (DBS) and microsampling technology

Dried blood spot (DBS) sampling and other microsampling techniques are gaining popularity due to their low invasiveness, simplicity of storage, and reduced transit needs. DBS approaches use just microliters of blood and are especially useful for large-scale epidemiological investigations, distant sample collection, and long-term monitoring. Future improvements to DBS extraction techniques and LC-MS/MS sensitivity are projected to broaden their use in forensic toxicology, occupational drug testing, and therapeutic drug monitoring.

### 7.4 High-resolution mass spectrometry for emerging NPS

The continued proliferation of NPS will require greater reliance on high-resolution mass spectrometry platforms such as Orbitrap and quadrupole time-of-flight (QTOF) instruments. Unlike traditional targeted approaches, HRMS allows retrospective data analysis, enabling laboratories to identify newly recognized compounds in previously acquired datasets. Future analytical workflows are likely to integrate untargeted HRMS screening with targeted LC–MS/MS confirmation, providing a flexible strategy capable of adapting to rapidly changing drug markets.

### 7.5 Green and sustainable analytical chemistry

Environmental sustainability will be a key factor in future technique development. The use of solvent-free extraction techniques, microextraction technologies, biodegradable sorbents, and miniaturized analytical platforms is predicted to grow significantly. Future analytical techniques will most likely be assessed not only on analytical performance but also on environmental effects, including greenness evaluation criteria such as AGREE, GAPI, AES, and NEMI.

## 8 Conclusion

The landscape of drug abuse detection is rapidly evolving in response to the dual challenges of traditional drug misuse and the emergence of NPS. While urine and blood remain the most commonly used specimens, hair and OF provide valuable complementary insights, particularly in long-term monitoring. Mass spectrometry-based methods continue to set the benchmark for accuracy and sensitivity, yet the growing emphasis on portability, speed, and field applicability has fueled innovation in electrochemical, spectroscopic, and biosensor technologies. The convergence of conventional laboratory techniques with novel, rapid, and user-friendly platforms offers a promising future for drug abuse detection. Ultimately, interdisciplinary efforts that combine analytical chemistry, forensic science, clinical toxicology, and public health perspectives will be crucial in developing robust strategies to mitigate the global impact of drug abuse. The principal innovation highlighted in this review is the two-tiered screening–confirmation paradigm, which combines the operational advantages of portable screening technologies with the analytical reliability of laboratory-based confirmatory methods, offering a future-ready framework for forensic and clinical drug detection.
