# Supplementary material for: Detection of drugs of abuse: evolving analytical strategies and emerging challenges
Source: Front Chem. 2026 Aug 3;14:1739599. doi: 10.3389/fchem.2026.1739599 (PMC13478270; doi:10.3389/fchem.2026.1739599)
Supplement: Supplementary file 1 [file Supplementaryfile1.docx]

**Systematic Literature Search Methodology**

1. **Methodology**

A systematic literature search methodology was employed for this review to ensure comprehensive coverage, analytical transparency, and reproducibility. The general principles of systematic scientific reviewing were adapted for the specific context of forensic and analytical toxicology research. The process involved four key stages: (1) literature search and identification, (2) screening and selection based on inclusion/exclusion criteria, (3) data extraction and synthesis, and (4) quality assessment.

**1.1. Literature Search Strategy**

A systematic search of the scientific literature was conducted to identify relevant peer-reviewed articles published between 2011 and 2025 This timeframe was selected to capture the most recent advancements in analytical toxicology, particularly the rise of new psychoactive substances (NPS). The primary electronic databases utilized were PubMed, Scopus, Web of Science, and Google Scholar. The search strategy combined keywords and Boolean operators (AND, OR) to target the core themes of the review. Sample search strings included:

- ("LSD" OR "lysergic acid diethylamide") AND ("detection" OR "analysis" OR "LC-MS" OR "GC-MS" OR "electrochemical sensor")
- ("synthetic cathinones" OR "bath salts" OR "cathinone derivatives") and ("forensic toxicology" OR "mass spectrometry" OR "biosensor")
- ("synthetic cannabinoids" OR "Spice" OR "K2") AND ("urine analysis" OR "hair analysis" OR "immunoassay" OR "colorimetric")
- ("psilocybin" OR "psilocin" OR "magic mushrooms") AND ("quantification" OR "HPLC" OR "forensic evidence")
- ("MDMA" OR "ecstasy" OR "3,4-methylenedioxymethamphetamine") and ("seized materials" OR "oral fluid" OR "portable device")
- ("methamphetamine" OR "crystal meth") AND ("on-site testing" OR "surface-enhanced Raman spectroscopy" OR "SERS")

The reference lists of key review articles and eligible primary studies were also manually screened to identify additional relevant publications that may have been missed in the database searches. The substances discussed were selected based on their high prevalence in forensic casework and the specific analytical challenges they present. While a significant emphasis is placed on New Psychoactive Substances (NPS), such as synthetic cannabinoids and cathinones, the review also encompasses widely used synthetic and semi-synthetic controlled substances, including LSD, MDMA, and methamphetamine. These "classic" synthetics were included because they remain primary targets for innovation in portable sensing and high-resolution mass spectrometry. Natural products like marijuana and cocaine were excluded to maintain a focused discussion on the evolving chemical profiles of synthetic and semi-synthetic drug classes. The selection of specific biological matrices, including blood, urine, hair, and oral fluid was determined by the pharmacokinetic properties and detection windows of the analytes. For example, hair analysis is prioritized for chronic exposure and retrospective monitoring of substances like MDMA, whereas oral fluid is highlighted for its utility in detecting recent impairment and its suitability for on-site electrochemical screening. Furthermore, the search strategy was designed to bridge the gap between clinical toxicology and forensic chemistry by including studies on both biological specimens and non-biological matrices. Consequently, this review provides a comprehensive overview of the analytical pipeline, detailing the detection of drugs in human fluids as well as in seized materials such as powders, tablets, herbal blends, and blotter papers.

**1.2. Inclusion and Exclusion Criteria**

Studies were assessed for eligibility based on the following pre-defined criteria:

**Inclusion Criteria:**

Studies were included if they:

- Described the development, optimization, or validation of analytical techniques for identifying illicit drugs or their metabolites in biological (e.g., blood, urine, hair, oral fluid) or non-biological (e.g., seized materials, blotters) matrices.
- Provided quantitative performance metrics, including limits of detection (LOD), limits of quantification (LOQ), accuracy, precision, recovery, and selectivity/specificity.
- Were published in English-language, peer-reviewed scientific journals.
- Focused on techniques with clear applicability to forensic, clinical, or environmental toxicology.

**Exclusion Criteria:**

The following were excluded:

- Articles with insufficient methodological detail or validation data.
- Conference abstracts, editorials, non-peer-reviewed reports, and unpublished theses.
- Studies that were solely behavioral, pharmacological, or clinical in nature without a primary focus on analytical method development or application.
- Duplicate publications or studies superseded by more comprehensive work from the same research group.

**1.3. Data Extraction**

Data was methodically extracted for every study that qualified in terms of:

- The analytical method used (e.g., spectroscopic platform, electrochemical sensor, GC-MS, LC-MS/MS).
- The matrices (blood, urine, saliva, hair, seized materials, etc.) were examined.
- Method performance metrics (reproducibility, precision, selectivity, LOD, and LQ).
- Reported benefits, drawbacks, and suitability for forensic use.

Data were tabulated and categorized by drug class (e.g., MDMA, methamphetamine, psilocybin, LSD, cathinones, and cannabinoids). Trends in matrix specificity, portability, and analytical capability were the main topics of comparative analysis.

**1.4. Quality Assessment**

The methodological robustness of each included study was critically evaluated. This assessment considered several factors:

- Validation Rigor: Adherence to internationally recognized validation guidelines (e.g., SWGTOX, FDA Bioanalytical Method Validation, EMA guidelines).
- Matrix Effect Evaluation: Whether and how the study addressed potential matrix interferences.
- Calibration Strategy: The use of appropriate internal standards and calibration models.
- Selectivity and Specificity: The demonstration of the method's ability to distinguish the target analyte from potentially interfering substances.

The relative merits and limitations of field-deployable techniques versus laboratory-based methods were evaluated to provide a balanced perspective on the current state of detection technologies. This critical appraisal aimed to ensure that the review's conclusions are based on analytically sound and forensically relevant data.
